# Risk of Incident Stroke among Vegetarians Compared to Nonvegetarians: A Systematic Review and Meta-Analysis of Prospective Cohort Studies

**DOI:** 10.3390/nu13093019

**Published:** 2021-08-29

**Authors:** Jing-Wun Lu, Luo-Hua Yu, Yu-Kang Tu, Hung-Yu Cheng, Li-Yu Chen, Ching-Hui Loh, Tai-Li Chen

**Affiliations:** 1 Department of Physical Medicine and Rehabilitation, Hualien Tzu Chi Hospital, Buddhist Tzu Chi Medical Foundation, Hualien 970, Taiwan; jingwunlu@gmail.com (J.-W.L.); hycheng@tzuchi.com.tw (H.-Y.C.); 2 Department of Medical Education, Taipei Tzu Chi Hospital, Buddhist Tzu Chi Medical Foundation, New Taipei City 231, Taiwan; 102311132@gms.tcu.edu.tw; 3 Institute of Epidemiology and Preventive Medicine, College of Public Health, National Taiwan University, Taipei 106, Taiwan; yukangtu@ntu.edu.tw; 4 Department of Dentistry, National Taiwan University Hospital and School of Dentistry, National Taiwan University, Taipei 106, Taiwan; 5 Library of Hualien Tzu Chi Hospital, Buddhist Tzu Chi Medical Foundation, Hualien 970, Taiwan; rani37jason@gmail.com; 6 School of Medicine, Tzu Chi University, Hualien 970, Taiwan; 7 Center for Aging and Health, Hualien Tzu Chi Hospital, Buddhist Tzu Chi Medical Foundation, Hualien 970, Taiwan; 8 Department of Medical Education, Medical Administration Office, Hualien Tzu Chi Hospital, Buddhist Tzu Chi Medical Foundation, Hualien 970, Taiwan

**Keywords:** vegetarian, vegan, plant diets, stroke, cerebrovascular disease, nutritional status and vegetarian, vegetarian and health

## Abstract

Vegetarian dietary patterns provide health benefits for cardiovascular health; however, the studies examining the association of vegetarian diets with stroke incidence showed inconsistent findings. We systematically evaluated the risk of incident stroke among vegetarians (diets excluding meat, poultry, fish, and seafood) compared among nonvegetarians. A systematic search of PubMed, EMBASE, Cochrane Library, and Web of Science was performed until 20 May 2021. Prospective cohort studies comparing the risk estimates for incident stroke between vegetarians and nonvegetarians were included. Of 398 articles identified in the database search, data from seven cohort studies (408 total stroke cases in 29,705 vegetarians and 13,026 total stroke cases in 627,728 nonvegetarians) were included. The meta-analysis revealed no significant association between adhering to the vegetarian dietary patterns and the risk of incident stroke (HR = 0.86; 95% CI = 0.67–1.11; I^2^ = 68%, *n* = 7). Subgroup analyses suggested that studies conducted in Asia and those with a mean baseline age of participants 50–65 years showed a lower risk of stroke in vegetarians. Moreover, no significant association between vegetarian diets and the risk of ischemic stroke (HR = 0.56; 95% CI = 0.22–1.42; I^2^ = 82%, *n* = 3) or hemorrhagic stroke (HR = 0.77; 95% CI = 0.19–3.09; I^2^ = 85%, *n* = 2) was found. To be conclusive, no strong relationship between vegetarian diets and the incidence of stroke was observed. Given the limited certainty of evidence from NutriGrade, future well-designed studies are warranted to provide solid evidence on this topic.

## 1. Introduction

Stroke is characterized as an acute injury to the central nervous system due to vascular damage, including cerebral infarction, intracerebral hemorrhage, and subarachnoid hemorrhage [1]. It is the second-leading cause of death globally, accounting for approximately 1 of every 20 deaths in the United States, despite the increased death rate of the ongoing coronavirus disease pandemic [2,3]. Stroke is also the most prevalent cause of functional disability and cognitive impairment, causing a considerable burden on society and patients’ families [4,5]. Therefore, the prevention of stroke by modifiable factors such as diet is crucial to the public health [6,7].

A vegetarian diet is defined as a diet that excludes the consumption of meat, poultry, fish, or seafood (MPFS) and may or may not include dairy and eggs [8]. The reported health benefits of this diet in cardiovascular diseases have made it popular, thereby leading to its recommendation in several guidelines [9,10]. Recently, efforts were made to evaluate the benefits of a vegetarian diet in stroke prevention; however, the results were controversial and were largely based on cross-sectional surveys or case control studies [11,12]. A previous meta-analysis indicated that a 200 g/day increase of vegetable consumption was associated with a 13% decrease in the risk of stroke [13]. In contrast, recent cohort studies did not find any association between a vegetarian diet and the risk of stroke [14,15]. The results from the EPIC Oxford study even reported a higher risk of stroke in vegetarians than in meat eaters [16]. As the study populations and sample sizes were heterogeneous in the current publications, a systematic review with a meta-analysis is warranted to provide a better understanding and draw conclusions. Therefore, this study aimed to provide updated evidence on the risk of incident stroke among vegetarians compared to among nonvegetarians by performing a systematic review and meta-analysis of the prospective cohort studies.

## 2. Materials and Methods

This systematic review and meta-analysis were performed following the Preferred Reporting Items for Systematic Reviews and Meta-Analyses [17] and Meta-Analyses of Observational Studies in Epidemiology guidelines [18]. The protocol for this study was registered on PROSPERO (registration number: CRD42021255150).

### 2.1. Data Sources and Literature Search

A comprehensive literature search of PubMed, EMBASE, Cochrane Library, and Web of Science was performed for publications evaluating the association between vegetarian diets and stroke from the inception of the databases to 20 May 2021. The search strategy was modified with the assistance of an experienced informationist, and its details are described in the Methods section in the Supplementary Materials. We did not impose any restrictions on language, geographic locations, or search filters. Additionally, we scrutinized the reference lists or bibliographies of reviews and relevant studies to extend our database search.

### 2.2. Study Selection and Outcomes

Two investigators (J.-W.L. and L.-H.Y.) independently selected reports from the electronic databases and resolved discrepancies by discussion. To minimize information and recall biases [19], only prospective cohort studies that fulfilled the following criteria were deemed eligible: (1) studies published as original articles; (2) those including an exposure group with participants adhering to vegetarian diets (diets not containing meat, poultry, fish, or seafood—namely, lacto-ovo-vegetarians); (3) those including a comparison group with participants following any form of nonvegetarian diets (diets containing either meat, poultry, fish, or seafood); and (4) study outcomes including the risk estimates of incident stroke—namely, studies that excluded patients with a history of stroke before the index date. If the result of the same cohort was reported in more than one report, the report with the longest follow-up length was enrolled. Case reports, editorials, review articles, and nonhuman studies that did not report original findings were excluded. Reports that applied a plant-based diet index without identification of a vegetarian diet status were excluded. Furthermore, reports that compared the adherence to a vegetarian diet, i.e., highest quantile to the lowest quantile of vegetable consumption, were excluded.

The primary outcome was total incident stroke. The secondary outcomes were incident ischemic stroke and incident hemorrhagic stroke [1].

### 2.3. Data Extraction and Risk of Bias Assessment

Two investigators (J.-W.L. and L.-H.Y.) independently collated data using a prespecified standardized form that included the following items: first author, publication year, country, study design, study sample size, demographic data of participants (age and sex), average follow-up length, dietary assessment, the definition of vegetarians and nonvegetarians, the definition of stroke, and the covariates. We also extracted the adjusted HRs with their standard errors. If the data were not sufficient, efforts were made to contact the corresponding authors to obtain the relevant information.

The methodological quality of these individual studies was assessed by two independent investigators (J.-W.L. and L.-H.Y.) according to the Newcastle-Ottawa Scale (NOS) [20]. Each study was assessed for the quality of selection, comparability of cohorts, and ascertainment of outcomes. Any disagreements in data extraction and risk of bias assessment were resolved by discussion with the other investigators (C.-H.L. and T.-L.C.).

### 2.4. Data Synthesis and Statistical Analyses

All statistical analyses were conducted using the Review Manager, version 5.4.1 (The Nordic Cochrane Centre, Cochrane Collaboration) and Stata, version 17 (Stata Corporation, College Station, TX, USA). The pooled hazard ratios (HRs) and the corresponding confidence intervals (CIs) were synthesized using the DerSimonian–Laird random-effects model based on an assumption of considerable clinical heterogeneity [21].

Between-study heterogeneity was evaluated using the I^2^ statistics, wherein an I^2^ >50% indicated substantial heterogeneity [22]. To determine whether certain study-level factors would influence the pooled results, several predefined subgroup analyses were conducted according to the mean age, geographic location, sex, body mass index (BMI), and dietary assessment. For testing the robustness of our primary analyses, we conducted a leave-one-out sensitivity analysis by omitting one study at a time. In addition, the publication bias was evaluated by inspecting funnel plots and using Egger’s linear regression test.

### 2.5. Certainty of Evidence Assessment

To quantify the certainty of evidence for the association between the vegetarian diet and stroke, the NutriGrade tool was applied [23]. Eight items were assessed in each outcome of interest, and a total score was generated. The results of each outcome were interpreted as very low (0 to <4 points), low (4 to <6 points), moderate (6 to <8 points), or high (8–10 points) certainty of the evidence.

## 3. Results

### 3.1. Search Results

The detailed selection process is illustrated in Figure S1 in the Supplementary Materials. A total of 398 unique reports were identified in the initial database search. After removing the duplicates, the titles and abstracts of the remaining reports were screened for eligibility. Of these, the full texts of nine reports were retrieved. Ultimately, seven cohort studies in four reports were included in the final meta-analysis [14,15,16,24]. A list of the excluded studies during the selection process is presented in Table S1 in the Supplementary Materials.

### 3.2. Characteristics of Included Studies

Table 1 outlines the characteristics of the included studies [14,15,16,24]. Seven prospective cohorts were reported in four reports, all published since 2019. A total of 866,110 participants, investigated between 1984 and 2020, were enrolled. A total of three studies were conducted in the USA [14], two in Taiwan [24], and two in the UK [15,16]. Most of the included studies used a food frequency questionnaire to distinguish a vegetarian from a nonvegetarian diet. Two reports used the baseline intake to assess the vegetarian status [15,16], while the other two reports considered longitudinal follow-up data [14,24]. The three studies in the report by Baden et al. had a follow-up length of more than 20 years [14]. Stroke was defined by the hospital-based records and International Classification of Diseases codes. All of the included studies confirmed the incidence of stroke, as individuals with a history of stroke at the baseline were excluded. Adjusted covariates of individual studies were presented in Table S2 in the Supplementary Materials.

### 3.3. Risk of Bias Assessment

The risk of bias among the included studies after critical appraisal using the NOS is summarized in Table S3 in the Supplementary Materials. None of the studies were rated with a high risk of bias. A total of four studies did not score in the representativeness of the exposed cohorts category, because they only enrolled health professionals and volunteers [14,24]. The average follow-up lengths in three studies were less than 10 years [15,16]. Overall, all of our included studies were considered to be of “high quality”, because they scored ≥7 points on the NOS.

### 3.4. Vegetarian Diet and Risk of Total Stroke

**Figure 1:**
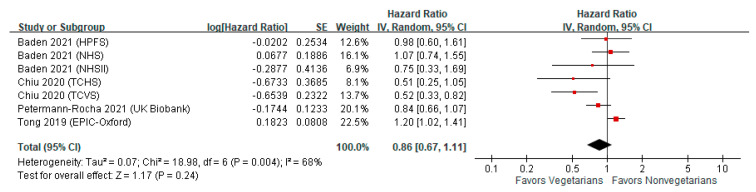
Forest plot showing the association of a vegetarian diet and the risk of incident stroke. The meta-analysis illustrated no association between a vegetarian diet and the risk of incident stroke. CI, confidence interval; IV, inverse variance; SE, standard error.

A total of seven studies (including 408 total stroke cases in 29,705 vegetarians and 13,026 total stroke cases in 627,728 nonvegetarians) reported the adjusted HRs of total stroke among vegetarians compared with nonvegetarians [14,15,16,24]. The meta-analysis showed that there was no significant association between vegetarian dietary patterns and the risk of incident stroke (HR = 0.86; 95% CI = 0.67–1.11; I^2^ = 68%, *n* = 7; Figure 1). 

### 3.5. Vegetarian Diet and Risk of Ischemic and Hemorrhagic Stroke

**Figure 2:**
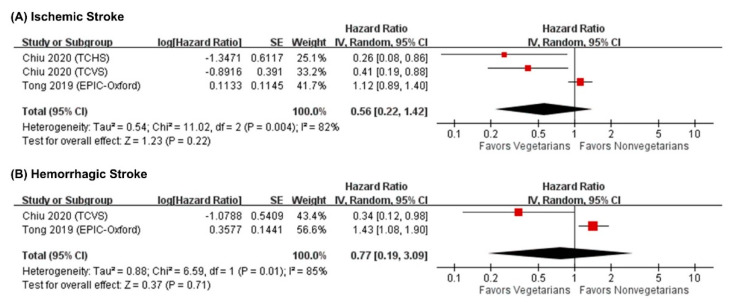
Forest plots showing the associations of a vegetarian diet and the risks of ischemic and hemorrhagic stroke. The meta-analysis showed that there was no association between a vegetarian diet and the risk of ischemic stroke or hemorrhagic stroke. CI, confidence interval; IV, inverse variance; SE, standard error.

A total of three studies (including 128 ischemic stroke cases in 54,034 participants) reported the adjusted HRs of ischemic stroke, while two studies (including 93 hemorrhagic stroke cases in 48,984 participants) reported that of hemorrhagic stroke comparing vegetarians with nonvegetarians. As shown in Figure 2, there was no significant association between vegetarian diets and the risk of ischemic stroke (HR = 0.56; 95% CI = 0.22–1.42; I^2^ = 82%, *n* = 3) or hemorrhagic stroke (HR = 0.77; 95% CI = 0.19–3.09; I^2^ = 85%, *n* = 2). 

### 3.6. Subgroup Analysis

Table 2 summarized the results of the subgroup analysis. Studies with a mean baseline age of participants between 50 and 65 years exhibited a lower risk of stroke in vegetarians than nonvegetarians (HR = 0.66; 95% CI = 0.45–0.95; I^2^ = 54%, *n* = 3). Studies conducted in Asia showed that vegetarians were at a lower risk of developing stroke than nonvegetarians (HR = 0.52; 95% CI = 0.35–0.76; I^2^ = 0%, *n* = 2), while those conducted in America (HR = 1.00; 95% CI = 0.76–1.32; I^2^ = 0%, *n* = 3) and Europe (HR = 1.02; 95% CI = 0.72–1.44; I^2^ = 83%, *n* = 2) did not show a significant difference in the risk of stroke between vegetarians and nonvegetarians. Moreover, studies that used the baseline intake to assess the vegetarian status showed a lower risk of stroke in vegetarians than nonvegetarians (HR = 0.66; 95% CI = 0.45–0.95; I^2^ = 54%, *n* = 3). Studies that considered longitudinal follow-up data to evaluate the vegetarian status showed a higher risk of stroke in vegetarians than nonvegetarians (HR = 1.15; 95% CI = 1.00–1.32; I^2^ = 0%, *n* = 4).

### 3.7. Sensitivity Analysis and Publication Bias

The leave-one-out sensitivity analysis demonstrated that the pooled HR was robust (Figure S2). Moreover, the potential publication bias was observed by inspecting the funnel plot in the Supplementary Materials (Figure S3) and using Egger’s linear regression test (*p* = 0.038).

### 3.8. Certainty of Evidence

The results of NutriGrade were demonstrated in Table 3. The summary score of each outcome showed that the overall certainty of evidence was low in total stroke, ischemic stroke, and hemorrhagic stroke. 

## 4. Discussion

To the best of our knowledge, this systematic review and meta-analysis of prospective cohort studies is the first to investigate the risk of stroke in vegetarians compared with nonvegetarians. We observed no significant association between vegetarian diets and the risk of incident stroke. Mean age, geographic locations, and a dietary assessment may be study-level factors to influence the pooled results. Overall, the certainty of evidence was low.

The results of our analyses may support the findings of previous studies. An umbrella review from 2020 concluded that vegetarian diets were not associated with a lower risk of stroke mortality [25]. Previous meta-analyses also demonstrated that vegetarian diets were not linked to cardiovascular and stroke mortality [26,27]. Our meta-analysis revealed no strong association between vegetarian diets and the incidence of stroke. On the contrary, vegetarian diets have been reported to have benefits for multiple health outcomes, such as a lower incidence of type 2 diabetes, coronary heart diseases, obesity, and greater life expectancy [26,28,29,30]. Besides, vegetarians are proven to have reduced cholesterol levels and cardiovascular diseases [25,31,32,33,34,35,36].

In nutritional studies comparing the effects of vegetarians with nonvegetarians, methodological limitations have arisen in multiple aspects similar to the comparison of high consumers versus low consumers. Instead, observational studies were designed to compare the difference between a group of 0 g/day consumers of MPFS to >0 g/day MPFS consumers, which may lose the dose–response effect. Previous dose–response meta-analyses indicated that the effect might vary greatly within the group of (any) consumers, and it might be nonlinear [37,38]. Therefore, no effect for vegetarians, as seen in our study, may be found if the study-level mean MPFS intake is low to moderate. The difference between the investigator-defined nonvegetarians may not consume enough MPFS to explicit a significant effect between vegetarians and nonvegetarians. These issues need to be covered by future research.

Another explanation for the findings may be the change in the composition of vegetarian diets over recent years. The vegetarian diet has experienced a nutritional transformation over the years [39], with high proportions of processed foods and refined sugars present in these diets [40]. As the consumption of unhealthy food was uncontrolled in most of our enrolled studies, this potential confounder may have an impact on the pooled estimates. In one of our enrolled studies, vegetarians were reported to consume more crisps, slices of pizza, and smoothie drinks than meat eaters [15]. 

Current research with participants adhering to a plant-based diet showed benefits in lowering the risks of stroke, and the results were consistent [14,41,42]. A vegetarian diet with a higher intake of unhealthy plant-based food (such as refined grains, added sugars, and fats) may be unlikely to yield maximal health benefits. Consequently, healthy plant-based food rather than a vegetarian diet may be a more decisive influence and determinant of the risk of stroke. The Mediterranean diet and Dietary Approaches to Stop Hypertension and other dietary patterns that emphasize plant foods have proven to be protectively associated with the incidence of stroke [7,43,44,45,46]. These diets do not suggest that one must abstain from MPFS; yet, the spirit of consuming healthy food is more important. 

Our subgroup analysis found that the incidence of total stroke was reduced in vegetarians aged 50–65 years. We postulated that a vegetarian diet might be associated with a lower body mass index, blood pressure, and glucose and cholesterol levels [26,34,35,36,47]. However, it cannot mitigate the inherent risk factors in young adults. There are still many risk factors, such as vasculopathy, cardiac defects, recent pregnancy, and other hypercoagulable states for stroke in young adults, defined as stroke patients aged younger than 50 years [48,49,50,51,52,53,54]. Furthermore, for older participants, vegetarian diets may lead to a deficiency of vitamin B_12_ and an increase of homocysteine, which may conversely increase the risk of stroke [55,56,57,58].

Another interesting finding of our subgroup analysis is that the incidence of total stroke was reduced among vegetarians in studies conducted in Asia but not in those conducted in the Western countries such as the USA and the UK. Environmental factors and lifestyle differences may partly explain this finding [59]. Further interethnic research may explore more details.

### Strength and Limitations

A key strength of this study is the large-scale study design, which included a total of 657,433 participants. We only included high-quality evidence and prospective cohort studies with long follow-ups to elucidate the temporal relationship between vegetarian diets and the risk of incident stroke. Moreover, we performed subgroup analyses and scored Nutri Grade to provide a better understanding on this topic.

The study has some limitations. First, several included studies that were conducted on the same study group. Hence, the generalizability of our study was limited. Second, as with most studies on vegetarian diets, the dose–response relationship was not elucidated in the included cohorts [16,27]. Investigating vegetarians as a continuity of adherence to a plant-based diet may facilitate the understanding of the effect of vegetarian diets on various health outcomes. Third, the results of two reports included in our review were based on baseline intake data only, which did not consider the longitudinal change in intake. More studies using follow-up data from ongoing cohorts may provide evidence regarding the length of adherence to vegetarian diets. Finally, there was evidence of publication bias. Studies with a higher risk of stroke in vegetarians were not published.

## 5. Conclusions

In summary, this study found no evidence of a strong relationship between vegetarian diets and incident stroke. Given the limited certainty of the evidence, the methodological recommendations made by this review might help future research teams better explore this topic. 

## Figures and Tables

**Table 1:** Characteristics of the included studies.

First Author, Year	Study Name (Country)	N of Participants	N of Cases (Absolute Rate of Stroke, %)	Age, Years, Mean (SD)	Female (%)	BMI, Mean (SD)	Ethanol Intake, g/day, Mean (SD)	Incidence of DM	Incidence of HTN	Average Follow-Up Length, Years (Date)	Dietary Assessment	Definition of Vegetarian/non-Vegetarians	Stroke Assessment	Covariate Adjustment Set	Risk Estimates, HR (95% CI)
Baden et al., 2021 [14]	NHS (US)	473 of vegetarians/65,987 of non-vegetarians	29 in vegetarians (6.1)/3575 in non-vegetarians	69 (7)	100	24.9 (5.2)	2.3 (7.2)	7%	42%	31.4 (1984–2016)	110-item FFQ, updated every 2–4 years	Lacto-ovo-vegetarians (meat and/or fish intakes were 0 or <1 serving per month)/meat and/or fish intakes were ≥1 serving per month	Hospital-based records	Age, race, smoking, alcohol intake, physical activity, total energy intake, DM, HTN, dyslipidemia, BMI, medication use	1.07 (0.74–1.55)
Baden et al., 2021 [14]	NHS II (US)	978 of vegetarians/90,539 of non-vegetarians	6 in vegetarians (0.6)/734 in non-vegetarians	48 (5)	100	24.8 (5.2)	3.1 (7.3)	2%	14%	25.8 (1991–2017)	110-item FFQ, updated every 2–4 years	Lacto-ovo-vegetarians (meat and/or fish intakes were 0 or <1 serving per month)/meat and/or fish intakes were ≥1 serving per month	Hospital-based records	Age, race, smoking, alcohol intake, physical activity, total energy intake, DM, HTN, hypercholesterolemia, BMI, medication use	0.75 (0.33–1.67)
Baden et al., 2021 [14]	HPFS (US)	320 of vegetarians/37,095 of non-vegetarians	16 in vegetarians (5)/1881 in non-vegetarians	68 (9)	0	25.0 (4.7)	4.5 (10.8)	8%	36%	26.0 (1986–2012)	110-item FFQ, updated every 2–4 years	Lacto-ovo-vegetarians (meat and/or fish intakes were 0 or <1 serving per month)/meat and/or fish intakes were ≥1 serving per month	Hospital-based records	Age, race, smoking, alcohol intake, physical activity, total energy intake, DM, HTN, hypercholesterolemia, BMI, medication use	0.98 (0.60–1.62)
Chiu et al., 2020 [15]	TCHS (Taiwan)	1424 of vegetarians/3626 of non-vegetarians	10 in vegetarians (0.7)/44 in non-vegetarians	53.8 (9.0)	59	23.0 (3.1)	8.1% ever drink alcohol	4.6%	14.2%	6.1 (2007–2014)	64-item FFQ, baseline	Lacto-ovo-vegetarians (consuming no meat and fish)/any consumption of meat or fish	ICD 9: 430–438	Age, sex, smoking, alcohol intake, betel nut, physical activity, education, DM, HTN, dyslipidemia, ischemic heart disease, and BMI	0.51 (0.25–1.06)
Chiu et al., 2020 [15]	TCVS (Taiwan)	2719 of vegetarians/5583 of non-vegetarians	24 in vegetarians (0.9)/97 in non-vegetarians	50.1 (9.3)	64	NA	11% ever drink alcohol	11.8%	9.4%	9.3 (2005–2014)	57-item FFQ, baseline	Lacto-ovo-vegetarians (consuming no meat and fish)/any consumption of meat or fish	ICD 9: 430–438	Age, sex, smoking, alcohol intake, betel nut, physical activity, education, DM, HTN, dyslipidemia, and ischemic heart disease	0.52 (0.33–0.82)
Petermann-Rocha et al., 2021 [16]	UK Biobank (UK)	7537 of vegetarians/415,304 of non-vegetarians	65 in vegetarians (0.9)/5881 in non-vegetarians	53.1 (7.9)	55	25.6 (4.6)	77.9% ever drink alcohol	NA	NA	8.5 (2006–2020)	24-hr recall questionnaire via Oxford WebQ, baseline	Lacto-ovo-vegetarians excluding vegans (consuming cheese and/or milk but not fish, poultry, or red meat)/meat-eaters (consumption of cheese, milk, fish, poultry, and red meat)	ICD 10: I60, I61, I63, I64	Age, sex, deprivation, race, smoking, alcohol intake, physical activity, and BMI	0.84 (0.66–1.07)
Tong et al., 2019 [24]	EPIC-Oxford (UK)	16,254 of vegetarians/31,934 of non-vegetarians	258 in vegetarians/814 in non-vegetarians	39.4 (13.1)	77	23	9.3 (12.8)	0.6%	5.8%	14 (1993–2016)	130-item FFQ, baseline and a follow-up 2010	Lacto-ovo-vegetarians including vegans (consuming no meat and fish)/meat-eaters (consumption of meat and fish)	ICD-9: 430–431, 433–434, 436ICD-10: I60–I61, I63–I64	Age, sex, smoking, alcohol intake, region, education, deprivation, physical activity, medication use	1.20 (1.02–1.40)

Abbreviation: BMI, body mass index; DM, diabetes mellitus; EPIC, European Prospective Investigation Into Cancer; FFQ, food frequency questionnaire; HR, hazard ratio; HPFS, Health Professionals Follow-Up Study; HTN, hypertension; ICD, International Classification of Disease; N, number; NA, not applicable; NHS, Nurses’ Health Study; SD, standard deviation; TCHS, Tzu Chi Health Study; TCVS, Tzu Chi Vegetarian Study; UK, United Kingdom; US, United States.

**Table 2:** Subgroup analysis.

Subgroups	No. of Studies	Hazard Ratio (95% CI)	I^2^ (%)
Overall	7	0.86 (0.67–1.11)	68
Mean age			
Age < 50 y	2	1.13 (0.83–1.54)	20
50 y ≦ Age < 65 y	3	0.66 (0.45–0.95) *	54
Age ≥ 65 y	2	1.04 (0.77–1.40)	0
Geographic locations			
America	3	1.00 (0.76–1.32)	0
Asia	2	0.52 (0.35–0.76) *	0
Europe	2	1.02 (0.72–1.44)	83
Sex			
Men	1	0.98 (0.60–1.61)	NA
Women	2	1.01 (0.72–1.41)	0
Body mass index (BMI) category			
Normal (18.5 ≦ BMI < 24.9)	4	0.98 (0.72–1.33)	52
Overweight (BMI ≥ 25.0)	2	0.87 (0.70–1.08)	0
Dietary assessment			
Baseline intake	3	0.66 (0.45–0.95) *	54
Longitudinal follow-up	4	1.15 (1.00–1.32) *	0

* *p* < 0.05. NA, not applicable.

**Table 3:** Item-level scoring for the NutriGrade tool and certainty of evidence for association between a vegetarian diet and stroke.

	Nutri Grade Items ^a^									
Comparison	Item 1 ^b^	Item 2 ^c^	Item 3 ^d^	Item 4 ^e^	Item 5 ^f^	Item 6 ^g^	Item 7 ^h^	Item 8 ^i^	Total Score	Certainty of Evidence
Total stroke	2	0	0.1	1	0	1	0	0	4.1	Low
Ischemic stroke	2	0	0.1	1	0	1	0	0	4.1	Low
Hemorrhage stroke	2	0	0.1	1	0	1	0	0	4.1	Low

^a^ Item-level NutriGrade tool scoring (points) [23]. ^b^ Item 1 = Risk of bias, study quality, and study limitations (0–2 points)—2 points if the mean Newcastle–Ottawa Score for a comparison ≧7 points. ^c^ Item 2 = Precision (0 to 1 point)—1 point if 500 events and 95% CI excluded null value, or otherwise, the 95% CI overlaps with the null value but excludes important benefit or harm (relative risk (RR) < 0.8 or >1.2). ^d^ Item 3 = Heterogeneity (0 to 1 point)—1 point if 10 studies, heterogeneity measures adequately reported, and no important heterogeneity found or otherwise subgroup/sensitivity analyses conducted. ^e^ Item 4 = Directness (0 to 1 point)—1 point if no important differences in the population or intervention; hard clinical outcomes. ^f^ Item 5 = Publication bias (0 to 1 point)—1 point if no evidence for publication bias with the test or plot (10 or more studies). ^g^ Item 6 = Funding bias (0 to 1 point)—1 point if the report from an academic or research institution. ^h^ Item 7 = Effect size (0–2 points)—1 point if HR < 0.80 to 0.50 or >1.20 to 2 and corresponding test statistically significant; 2 points if HR < 0.5 or >2.0 and the corresponding test statistically significant (highest versus lowest category). ^i^ Item 8 = dose-response (0 to 1 point)—1 point if a significant linear/nonlinear dose-response relation.

## Data Availability

The data was already published in the databases.

## Supplementary Materials

The following are available online at https://www.mdpi.com/article/10.3390/nu13093019/s1: Methods: Detailed search strategy modified to accommodate different databases. Figure S1. PRISMA flowchart of the selection process. Figure S2. Leave-one-out sensitivity analysis. Figure S3. Funnel plot for total stroke. Table S1. List of excluded studies during the selection process. Table S2. Adjusted covariates of individual studies. Table S3. Risk of bias assessment of the included studies using the Newcastle–Ottawa Scale.
