# Supplementary material for: Risk of Incident Stroke among Vegetarians Compared to Nonvegetarians: A Systematic Review and Meta-Analysis of Prospective Cohort Studies
Source: Nutrients. 2021 Aug 29;13(9):3019. doi: 10.3390/nu13093019 (PMC8471883; doi:10.3390/nu13093019)
Supplement: Supplementary file 1 [file nutrients-13-03019-s001.zip › nutrients-1319341-supplementary.pdf]

Supplemental Material

Methods. Detailed search strategy modified to accommodate different databases

Figure S1. PRISMA flowchart of the selection process

Table S1. List of excluded studies during selection process

Table S2: Adjusted covariates of individual studies

Table S3. Risk of bias assessment of the included studies using the Newcastle-Ottawa Scale

Figure S2. Leave-one-out sensitivity analysis

Figure S3. Funnel plot for total stroke

Methods. Detailed search strategy modified to accommodate different databases

PubMed

("Diet, Vegetarian"[mesh] OR "Vegetarian Diet"[tw] OR "Vegan Diet"[tw] OR "Plant-based Diet"[tw])

AND

("Stroke"[mesh] OR Stroke[tw] OR Cerebrovascular*[tw])

Embase

(vegetarian$ OR vegan$ OR plant-based$).mp. OR ((vegetarian adj3 diet*) OR (vegan adj3 diet*) OR (plant-based adj3 diet*)).mp. OR exp "vegetarian"/ OR exp "vegetarian diet"/ OR exp "vegan"/ OR exp "vegan diet"/ OR exp "plant-based"/ OR exp "plant-based diet"/

AND

("stroke" OR cerebrovascular* OR "apoplexy").mp.

Cochrane Library

(MeSH descriptor: [Diet, Vegetarian] explode all trees OR "Vegetarian Diet" OR "Vegan Diet" OR "Plant-based Diet")

AND

(MeSH descriptor: [Stroke] explode all trees OR (Stroke OR Cerebrovascular* OR Apoplexy))

Web of science

ALL = ("Vegetarian Diet" OR "Vegan Diet" OR "Plant-based Diet")

AND

ALL = (Stroke OR Cerebrovascular* OR Apoplexy)

Summary of database search

PubMed: 78

Embase: 279

Cochrane Library: 12

Web of Science: 29

Figure S1. PRISMA flowchart of the selection process

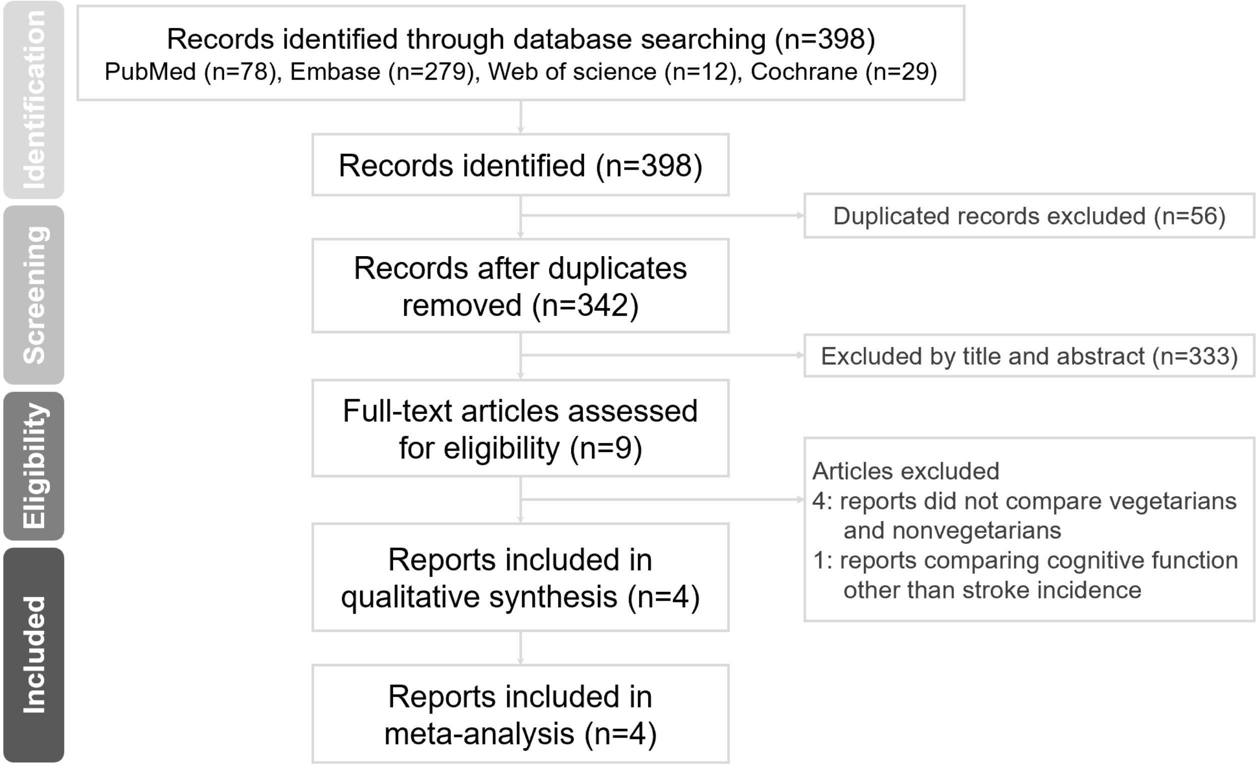

Table S1. List of excluded studies during selection process

Reference number	First author, year	Exclusion reason
[1]	Heianza etal. 2020	Did not compare vegetarians and nonvegetarians
[2]	Judd et al. 2013	Did not compare vegetarians and nonvegetarians
[3]	Mangat et al. 2013	Did not compare vegetarians and nonvegetarians
[4]	Pearson et al. 2016	Compare cognitive function but not stroke incidence
[5]	Shan et al. 2020	Did not compare vegetarians and nonvegetarians

[1] Heianza, Y., Zhou, T., Sun, D., Hu, F.B., Manson, J.E., Qi, L. Genetic susceptibility, plant-based dietary patterns, and risk of cardiovascular disease. *Am J Clin Nutr* **2020**, *112(1)*, 220-228.

[2] Judd, S.E., Gutiérrez, O.M., Newby, P.K., et al. Dietary patterns are associated with incident stroke and contribute to excess risk of stroke in black Americans. *Stroke* **2013**, *44(12)*, 3305-3311.

[3] Mangat, A., Grewal, D., Kaur, P., Jyotsna, R., Singh, R., Pandian, J.D. Dietary patterns in stroke patients in Northwest India. *Nutr Neurosci* **2013**, *16(6)*, 288-292.

[4] Pearson, K.E., Wadley, V.G., McClure, L.A., Shikany, J.M., Unverzagt, F.W., Judd, S.E. Dietary patterns are associated with cognitive function in the REasons for Geographic And Racial Differences in Stroke (REGARDS) cohort. *J Nutr Sci* **2016**, *5*, e38.

[5] Shan, Z., Li, Y., Baden, M.Y., et al. Association Between Healthy Eating Patterns and Risk of Cardiovascular Disease. *JAMA Intern Med* **2020**, *180(8)*, 1090-1100.

Table S2: Adjusted covariates of individual studies

First author, year	Study name (Country)	Age	Sex	Smoking	BMI	Alcohol	Ethnicity	Physical activity	Total energy intake	Aspirin	DM	HTN	Dyslipidemia	Education
Baden et al. 2021	NHS (US)	•		•	•	•	•	•	•	•	•	•	•	
Baden et al. 2021	NHS II (US)	•		•	•	•	•	•	•	•	•	•	•	
Baden et al. 2021	HPFS (US)	•		•	•	•	•	•	•	•	•	•	•	
Chiu et al. 2020	TCHS (Taiwan)	•	•	•	•	•		•			•	•	•	•
Chiu et al. 2020	TCVS (Taiwan)	•	•	•		•		•			•	•	•	•
Petermann-Rocha et al. 2021	UK Biobank (UK)	•	•	•	•	•	•	•			•	•	•	
Tong et al. 2019	EPIC-Oxford (UK)	•	•	•	•	•		•			•	•	•	•

Abbreviations: DM, diabetes mellitus; HTN, hypertension, UK, United Kingdom; US, United States.

Table S3. Risk of bias assessment of the included studies using the Newcastle-Ottawa Scale

	**Selection**				**Comparability**		**Outcome**			**Total Quality Score**
Source, study name (Country)	Representativeness of exposed cohort	Selection of non- exposed cohort	Ascertainment of exposure	The outcome of interest was not present in the start of the study	Adjusted for age/sex	Adjusted for comorbidities	Assessment of outcome	Follow-up length	Loss to follow-up rate	
Baden et al. 2021, NHS (US)	0[a]	1	1	1	1	1	1	1	1	**8**
Baden et al. 2021, NHS II (US)	0[a]	1	1	1	1	1	1	1	1	**8**
Baden et al. 2021, HPFS (US)	0[a]	1	1	1	1	1	1	1	1	**8**
Chiu et al. 2020, TCHS (Taiwan)	1	1	1	1	1	1	1	0	1	**8**
Chiu et al. 2020, TCVS (Taiwan)	0[b]	1	1	1	1	1	1	0	1	**7**
Petermann-Rocha et al. 2021, UK Biobank (UK)	1	1	1	1	1	1	1	0	1	**8**
Tong et al. 2019, EPIC-Oxford (UK)	1	1	1	1	1	1	1	1	1	**9**

[a]Baden *et al*. recruited nurses and health care professionals. Selected groups might cause potential risk of bias in the representativeness of exposed cohort.

[b]The TCVS cohort in study by Chiu et al.[22] consisted of volunteers from Tzu Chi sites. Selected groups might cause potential risk of bias in the representativeness of exposed cohort.

Selection:

1) Representativeness of exposed cohort: 1, truly or somewhat representative of a community/population-based study; 0, selected group of users or lack of description of the derivation of the

cohort.

2) Selection of non-exposed cohort: 1, drawn from the same community as the exposed cohort; 0, drawn from a different source or lack of description of the derivation of the non-exposed cohort.

3) Ascertainment of exposure: 1, validation of the questionnaire of vegetarian definition with secure record or structured interview; 0, written self-report or lack of description of validation of the questionnaire of vegetarian definition.

4) Demonstration that outcome of interest was not presented at start of study: 1, yes; 0, no.

Comparability:

1) Study adjusted for age and sex: 1, yes; 0, no.

2) Study adjusted for any comorbidities: 1, yes; 0, no.

Outcome:

1) Assessment of outcome: 1, independent blind assessment, confirmed by medical records or record linkage; 0, self-reported or no description.

2) Was follow-up long enough for outcomes to occur: 1, mean/median duration of follow-up at least ten years; 0, mean/median duration of follow-up < ten years.

3) Loss to follow-up rate: 1, complete follow-up or loss to follow-up rate less than 20%; 0, loss to follow-up rate more than 20% or no statement.

Abbreviations: EPIC, European Prospective Investigation Into Cancer; HPFS, Health Professionals Follow-Up Study; NHS, Nurses' Health Study; TCHS, Tzu Chi Health Study; TCVS, Tzu Chi Vegetarian Study; UK, United Kingdom; US, United States.

Figure S2. Leave-one-out sensitivity analysis

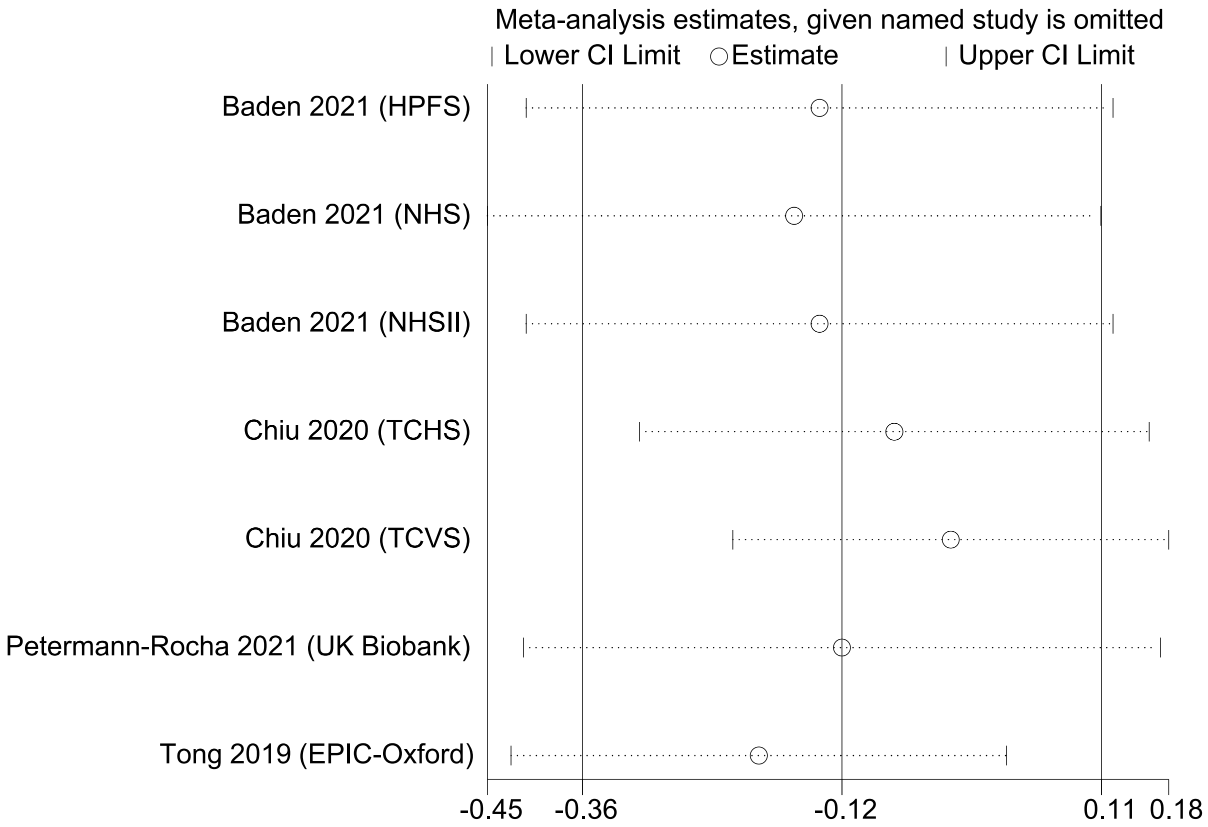

Abbreviations: CI, confidence interval; EPIC, European Prospective Investigation Into Cancer; HPFS, Health Professionals Follow-Up Study; NHS, Nurses' Health Study; TCHS, Tzu Chi Health Study; TCVS, Tzu Chi Vegetarian Study.

Figure S3. Funnel plot for total stroke

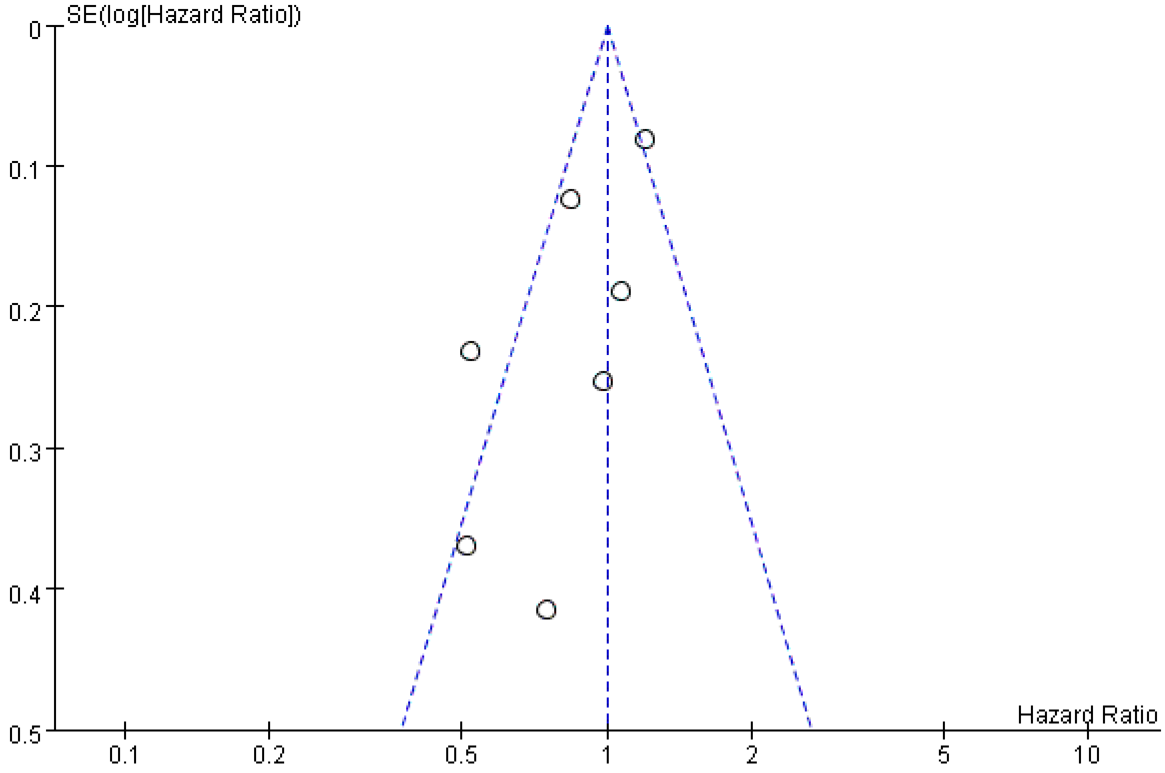

Abbreviations: SE, standard error.
